# Intention to consume 3D-printed food: perspectives across generational divides

**DOI:** 10.3389/fnut.2026.1731967

**Published:** 2026-04-01

**Authors:** Nicole Cunningham, Adeyemi Ayotunde Adeyanju, Oluwafemi Ayodeji Adebo

**Affiliations:** 1 Department of Marketing Management, College of Business and Economics, University of Johannesburg, Johannesburg, South Africa; 2 Centre for Innovative Food Research (CIFR), Department of Biotechnology and Food Technology, Faculty of Science, University of Johannesburg, Doornfontein Campus, Johannesburg, South Africa

**Keywords:** 3D-printed food, awareness, food neophobia, Generation X, Generation Y

## Abstract

As 3D food printing continues to emerge as a transformative food technology, understanding consumer acceptance across generational divides is critical for its successful adoption. However, existing research on 3D-printed food has largely assumed homogeneous behavioral mechanisms across age groups, offering limited theoretical insight into how generational context moderates consumer acceptance of this emerging food technology. This study examines the intention to consume 3D-printed food among the 30- to 59-year-old South African demographic, with a focus on the generational spans of Generation X and Generation Y. A quantitative, descripto-explanatory approach was employed, involving participants with some knowledge of 3D-printed food. Data was gathered through a self-administered online questionnaire, and a total of 355 complete responses were collected. Survey constructs were based on validated scales and included awareness, perceived benefits, food neophobia, food familiarity, attitude, health consciousness, social norms, convenience orientation, capacity, autonomy, and intention. The reliability and validity of the measurement model were evaluated using SmartPLS. Reliability and validity tests confirmed strong internal consistency and construct validity. Comparisons across generations indicated that awareness, perceived benefits, and attitude were important predictors of intention in both groups. Food neophobia had a negative effect on both groups, and social norms affected intention only in Generation Y. Health consciousness was a significant predictor of attitude for Generation X, but not for Generation Y. More traditional drivers of food choice, like familiarity, convenience, capacity, and autonomy, showed very weak effects. Finally, attitude was the strongest predictor of intention across both generations. Additionally, these results highlight significant differences between generations and offer valuable insights for developing targeted communication strategies to facilitate the dissemination of 3D-printed food technologies.

## 1 Introduction

Shifts in the global food market are unfolding due to technological advancements, accompanied by the demand for more sustainable, specialized, and creative food products (1). One of the newest innovations, the 3D printing of food, is gaining popularity and attention in the field of food technology, as it has the potential to change the ways food is produced and consumed (2, 3). 3D food printing can develop edible structures, layer-by-layer, thus enabling the customization of the nutritional content, shape, texture, and esthetics with precision (1, 4). The potential that 3D printed food offers is useful in tackling multiple issues, such as reducing food waste, providing tailored nutrition, texture modification (particularly beneficial for elderly or dysphagia patients), and creating innovative sensory experiences (5, 6).

Although technological advancements have significantly enhanced the development of 3D-printed food, its successful integration into mainstream consumer markets is largely contingent upon public acceptance (7). Previous studies have demonstrated that consumer attitudes toward novel food technologies are shaped by multiple factors, including perceived benefits and risks, food neophobia, and cultural factors (3, 8). Like other emerging food technologies, such as genetically modified organisms, cultured meat, and edible insects, 3D printed food can elicit ambivalent responses stemming from perceptions of unfamiliarity with technology and ethical considerations (9, 10). Furthermore, generational differences play a significant role in shaping acceptance, as attitudes toward food innovations are often influenced by distinct sociohistorical contexts, technological exposure, and lifestyle orientations (11, 12). This is supported by Rogers (13), who states that technology adoption patterns differ across different generational cohorts. Commonly recognized generational cohorts include the Baby Boomer generation (born 1946–1964), Generation X (born 1965–1979), Generation Y or Millennials (born 1980–1994), and Generation Z (born after 1995), also referred to as Generation C (“connected”) or the post-millennial generation (14). For example, younger consumers (e.g., Generation Z and Millennials) tend to be more open to technologically-driven and sustainability-focused food innovations, whereas older generations are generally more cautious or skeptical (12, 14).

Existing research (3, 7, 8, 15, 16) shows that consumer acceptance of 3D-printed food is influenced by factors such as awareness, perceived benefits, food neophobia, attitudes, health considerations, and social norms, with most studies reporting these relationships at an aggregate level across consumer populations. However, it remains unclear whether these behavioral drivers operate uniformly across generational cohorts, particularly in emerging-market contexts where socio-cultural conditions, technological exposure, and food norms differ markedly. Most of these studies have largely assumed homogeneous psychological mechanisms across age groups, with limited empirical examination of generation-specific pathways shaping intention to consume 3D-printed food. Addressing this gap, the present study explicitly models generational variation in the psychological determinants of intention to consume 3D-printed food by comparing Generation X and Generation Y consumers in South Africa, thereby revealing how and why the relative influence of key drivers differs across cohorts and extending food-technology acceptance frameworks through a generationally moderated perspective.

## 2 Research methodology

### 2.1 Research design and theoretical framework

**FIGURE 1:**
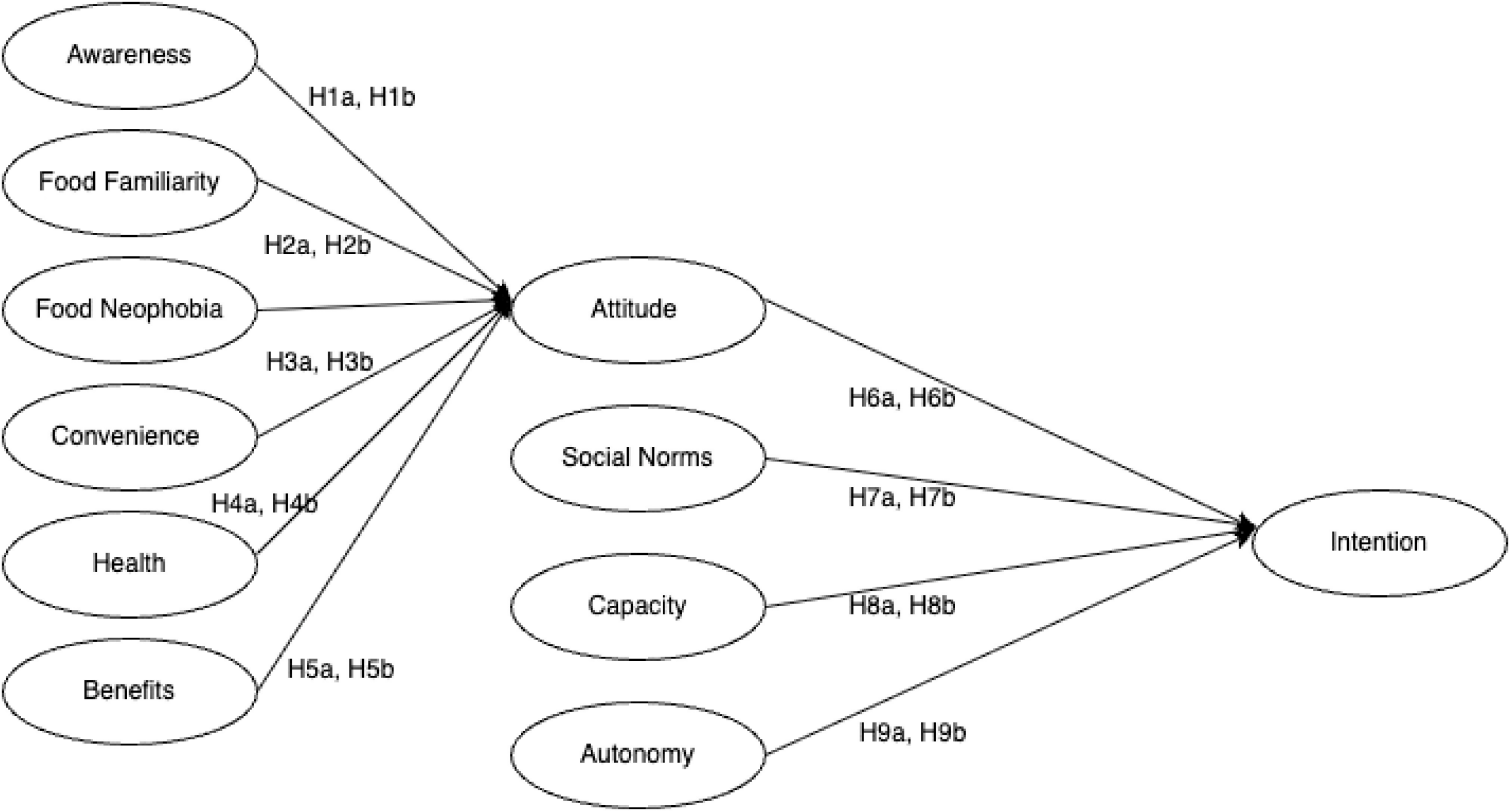
Conceptual model representing the relationships predicted to influence the intention of consuming 3D-printed food.

This study adopted a quantitative, descripto–explanatory research design to examine the psychological mechanisms underlying consumers’ intention to consume 3D-printed food across generational cohorts in South Africa. This design is appropriate for simultaneously describing key consumer characteristics and testing theoretically grounded relationships among latent constructs (17), as depicted in Figure 1. Ethical clearance for the study was obtained from the Faculty of Science Ethics Committee at the University of Johannesburg (Approval Reference: 2023-05-03/Adebo).

The conceptual framework is theoretically anchored in the Theory of Planned Behavior (TPB) (18), which serves as the central explanatory backbone of the model. TPB is particularly suited to the study of emerging food technologies, as it explicitly links belief-based evaluations and social influence to behavioral intention under conditions of uncertainty and novelty. Within this framework, attitude toward 3D-printed food and intention to consume represent the core outcome constructs. In the context of 3D-printed food, intention to consume goes beyond a routine purchase decision; it reflects a technology adoption judgment situated at the intersection of food choice, innovation evaluation, and risk appraisal. Behavioral intention theory conceptualizes intention as the immediate motivational antecedent of behavior (19). However, when the focal behavior involves a technologically mediated and symbolically unfamiliar food product, intention formation is additionally shaped by mechanisms identified in technology acceptance and novel food adoption research. Technology acceptance frameworks (e.g., TAM and UTAUT) emphasize that adoption decisions are guided by evaluations of usefulness, ease of use, and social influence, while food innovation literature highlights the roles of food neophobia, perceived risk, perceived naturalness, and trust in food systems. Accordingly, the present study positions intention to consume 3D-printed food as the outcome of a structured cognitive appraisal process integrating innovation beliefs, value orientations, social norms, and perceived control, rather than as a purely descriptive dependent variable.

Consistent with TPB logic, belief-related constructs—awareness, perceived benefits, health consciousness, and food neophobia—are modeled as antecedents shaping attitudinal evaluations. Recent empirical work on 3D-printed food adoption further substantiates the inclusion of these belief-based antecedents. Drawing on qualitative insights from professional chefs, Abdul Latip et al. (20) identify technological uncertainty, sensory concerns, economic feasibility, and socio-cultural resistance as central challenges shaping acceptance of 3D-printed food in commercial settings. Although examined from a supply-side perspective, these challenges translate directly into consumer-level belief structures, particularly perceived benefits, food neophobia, and social acceptance. Their findings reinforce the theoretical relevance of modeling belief-driven evaluations as precursors to attitude formation when food technologies are novel, unfamiliar, and symbolically contested.

Social norms capture the normative influence component of TPB, while capacity and autonomy reflect perceived behavioral control, representing consumers’ perceived ability and freedom to adopt 3D-printed food. This positioning ensures conceptual coherence by aligning all structural paths with TPB’s foundational assumptions regarding intention formation. While TPB serves as the primary theoretical framework, complementary perspectives are incorporated to contextualize belief formation in a novel food environment. Diffusion of Innovation theory (21) informs the inclusion of awareness by explaining how exposure to technological innovation influences early-stage acceptance. Similarly, the Value–Attitude–Behavior (VAB) hierarchy (22) provides insight into how underlying consumption values—such as health orientation and openness to innovation—translate into attitudes toward unfamiliar foods. Importantly, these perspectives are employed as supporting lenses rather than independent theoretical drivers, reinforcing TPB’s role as the unifying framework.

The suitability of the Theory of Planned Behavior as the core explanatory framework in the context of 3D-printed food is further supported by recent comparative modeling evidence. Kamrath et al. (23) empirically tested a sector-specific Food Technology Acceptance Model (FTAM) alongside the generic TPB to explain consumers’ intention to consume 3D-printed food. Their findings demonstrate that TPB retains strong explanatory power even in this highly novel and technology-mediated food context, with intention significantly shaped by belief-based evaluations such as perceived benefits and perceived risks, subjective knowledge, institutional trust, and subjective norms. Importantly, the authors show that these technology-specific beliefs do not operate independently of TPB but instead align closely with its core assumption that evaluative and normative processes are proximal drivers of intention. This evidence provides robust theoretical justification for modeling awareness-related knowledge, perceived benefits, and social norms as antecedents of intention through attitudinal pathways within a TPB-based framework.

Beyond cognitive evaluations, Kamrath et al. (23) further demonstrate that intention formation toward 3D-printed food is conditioned by trust dynamics, particularly affective trust in the food industry, which shapes perceived information credibility and subsequent rational evaluations. Their findings indicate that information exposure alone is insufficient to influence intention unless filtered through pre-existing trust structures. This supports the present study’s theoretical assumption that belief-based constructs—such as awareness and perceived benefits—are interpreted through broader evaluative frameworks rather than exerting direct, uniform effects on intention, particularly in contexts characterized by technological uncertainty and low consumer familiarity.

At a broader systems level, the perceived benefits of 3D-printed food are increasingly shaped by macroeconomic and structural narratives surrounding food production. Niyet et al. (24) conceptualize 3D food printing as a strategic response to labor scarcity, highlighting its potential to enhance efficiency, enable personalized nutrition, and support sustainable food systems. Such narratives contribute to consumer awareness and benefit perceptions by framing 3D-printed food not merely as a technological novelty but as a functional solution to contemporary food system challenges. These contextual drivers are particularly relevant in generational analyses, as younger cohorts tend to be more receptive to innovation framed around sustainability, efficiency, and technological progress.

Recent food-consumption research further supports the Theory of Planned Behavior (TPB) as a suitable core framework for explaining intention formation in novel and technology-mediated food contexts. Chakraborty and Dash (25), applying the Theory of Consumption Values (TCV) to natural food products, demonstrate that functional, emotional, epistemic, and conditional values shape purchase intention primarily through evaluative mechanisms, such as attitudes, rather than exerting uniform, direct effects. Their findings underscore that consumer intention toward food innovations is driven by value-based cognitive and affective appraisals, reinforcing TPB’s emphasis on attitudes as a proximal determinant of intention. Complementarily, Suhud et al. (26) show that in digitally mediated food-related commerce, intention formation is highly context-dependent, with social and marketing stimuli operating through trust, credibility, and normative influence rather than stable preferences alone. Together, these studies highlight that behavioral drivers of food choice are not universal but contingent on product novelty, consumption context, and consumer characteristics. Building on this evidence, the present study conceptualizes generation as a theoretical moderator within the TPB framework, conditioning the relative salience of attitudinal beliefs, social norms, and health-related evaluations in shaping intention to consume 3D-printed food.

Thus, the proposed framework extends established behavioral intention theory by embedding food technology acceptance constructs within a generational cohort lens, offering a theoretically grounded explanation of how innovation-related beliefs translate into consumption intention across socio-historically distinct consumer groups.

### 2.2 Generational cohort as a theoretical moderator

Generational cohort theory provides a sociological and consumer psychology foundation for understanding heterogeneity in innovation adoption. Generations represent groups of individuals socialized during distinct historical, technological, and economic periods, leading to enduring differences in cognitive schemas, risk perception, value priorities, and openness to emerging technologies. In consumer behavior research, cohort effects have been shown to influence technological readiness, trust in institutions, reliance on social influence, and sensitivity to uncertainty—factors that are particularly salient in evaluating novel food technologies (27). Because 3D-printed food combines technological innovation with food-related risk considerations, generational membership is theoretically expected to condition how consumers interpret perceived benefits, health concerns, social norms, and control beliefs in forming intention. Rather than treating generational cohort as a purely descriptive segmentation variable, this study conceptualizes generation as a theoretical moderator within the TPB framework. Prior research demonstrates that consumption values, risk perceptions, and reliance on social influence vary systematically across age cohorts due to differences in life stage, technological socialization, and value prioritization (11). These generational differences suggest that the relative importance of TPB components—attitude, subjective norms, and control perceptions- may vary across population segments.

Within the context of novel food technologies, younger consumers who have grown up amid rapid technological advancement may rely more heavily on social cues, perceived benefits, and innovation-related beliefs, while older consumers may place greater emphasis on health considerations and risk reduction. Accordingly, this study theorizes that generation moderates the strength of TPB pathways, influencing how beliefs translate into attitudes and how attitudes and norms shape intention. To empirically test these moderated relationships, multigroup analysis (MGA) is employed to compare structural paths between Generation X and Generation Y, allowing the study to move beyond surface-level comparison toward a theory-driven explanation.

Thus, rather than presenting a standalone empirical comparison, the proposed model extends established behavioral intention theory by embedding food technology acceptance constructs within a generational cohort framework, offering a theoretically grounded explanation of how innovation-related beliefs translate into consumption intention across socio-historically distinct consumer groups.

### 2.3 Sampling, data collection, and measurement

The research targeted South African consumers aged 30–59 who were familiar with the concept of 3D-printed food. To access relevant participants, purposive sampling was applied by requiring that respondents needed to meet two specific criteria: (1) that they were aged between the ages of 30 and 59 (Generation X and Y) and (2) that they had awareness of 3D-printed food (possibly through news reports, documentaries, and television programs highlighting food innovation; as well as social media platforms). Purposive sampling was intentionally adopted to ensure the study captured informed perspectives from individuals within the target generational groups (Generation X and Y) and with at least some awareness of 3D-printed food, as this foundational familiarity is essential for forming meaningful opinions about the intention to consume food from an emerging technology. Given the novelty of 3D-printed food in South Africa and the generally low public exposure, including respondents with no awareness, would have resulted in uninformed or speculative responses, thereby weakening the validity of the constructs being measured. Also, the study focused specifically on respondents aged 30–59 to capture the generational perspectives of Generation X and Generation Y, who represent the demographic groups most actively engaged in household food purchasing, decision-making, and adoption of emerging food technologies. Younger Generation Z consumers (under 30) often have limited independent purchasing power and less stable food-related behavior patterns, making them less suitable for assessing sustained consumption intentions. Conversely, Baby Boomers (60+) typically demonstrate lower openness to novel food technologies and reduced likelihood of adopting innovations such as 3D-printed foods, as documented in prior research on technology acceptance across age groups (11). Focusing on the 30–59 age cohort, therefore, allowed the study to target the population segment most relevant to understanding realistic market uptake and generational differences in intention to consume 3D-printed food.

Data collection was facilitated through a self-administered online questionnaire hosted on Google Forms and distributed via social media platforms. At the beginning of the survey, a brief introduction explained the study’s purpose and outlined relevant ethical considerations. Two screening questions were used to determine eligibility: one assessed respondents’ familiarity with 3D-printed food, and the other confirmed age compliance. The first question was especially important due to the limited public exposure to this technology.

Following the screening, the questionnaire collected demographic information, including age, gender, race, education level, employment status, income, and province of residence. The core section of the questionnaire included items related to the study’s eight constructs, each measured on a 7-point Likert scale (1 = strongly disagree to 7 = strongly agree). These constructs are visualized in Figure 1, and the corresponding measurement items are listed in Table 1. Measurement scales were adapted from prior studies: constructs such as awareness, benefits, food familiarity, food neophobia, attitude, and social norms were based on Ng et al. (15), convenience and health were derived from Brunner et al. (16) while capacity and autonomy were adapted from Armitage and Conner (18) and lastly, intention adapted from Soh et al. (28). A total of 355 valid responses were collected. A response was considered valid if the participant met the screening criteria and completed the entire questionnaire. This theoretically grounded measurement strategy ensures alignment between TPB constructs and observed variables, enabling robust testing of both direct effects and generational moderation within the proposed framework.

**TABLE 1:** Reliability (Cronbach alpha and composite reliability) and convergent validity (AVE) of each construct and its corresponding items.

Construct and items	Factor loadings	α	CR	AVE
Awareness				
I have already heard or read about 3D-printed food.	0.803	0.795	0.807	0.514
I roughly know how the 3D printing process works.	0.791			
I have consumed 3D-printed food before.	0.717			
I have dealt with 3D-printed food before.	0.742			
Benefits				
3D food printing can produce food easier and more conveniently.	0.883	0.911	0.928	0.618
3D food printing can produce food to meet the dietary requirements of people with health issues.	0.715			
3D food printing can design food with personalized nutritional needs.	0.714			
3D food printing can help reduce food wastage.	0.789			
I have no trouble consuming 3D-printed food because it is safe to consume.	0.825			
I have no trouble consuming 3D-printed food because it is beneficial to my health.	0.836			
I have no trouble consuming 3D-printed food because it is a fun experience.	0.828			
I have no trouble consuming 3D-printed food because it is not disgusting.	0.836			
Food familiarity				
It is important to me that the food to be eaten is usually what I would eat.	0.904	0.832	0.899	0.748
It is important to me that I am familiar with the food I would eat.	0.876			
It is important to me that the food I eat is similar to the food I ate during my childhood.	0.813			
Food neophobia				
I am afraid to eat food that I have never eaten before.	0.766	0.898	0.920	0.622
I will not try a new food if I do not know what is in it.	0.753			
I do not trust new food.	0.806			
I do not like trying out new and different food.	0.819			
I do not need new food technology because there is already much tasty food around.	0.793			
The benefits of new food technology are always overstated.	0.769			
I will not try out high-tech food products because the ones I eat are already good enough.	0.786			
Convenience				
The less physical energy I need to prepare a meal, the better.	0.892	0.937	0.952	0.799
The ideal meal can be prepared with little effort.	0.908			
Preferably, I spend as little time as possible on meal preparation.	0.917			
I want to spend as little time as possible cooking.	0.906			
At home, I preferably eat meals that can be prepared quickly.	0.843			
Health				
It is important to me that the food I eat on a typical day contains a lot of vitamins and minerals.	0.871	0.945	0.956	0.784
It is important to me that the food I eat on a typical day keeps me healthy.	0.910			
It is important to me that the food I eat on a typical day is nutritious.	0.884			
It is important to me that the food I eat on a typical day is high in protein.	0.901			
It is important to me that the food I eat on a typical day is good for my skin, teeth, hair, nails, etc.	0.883			
It is important to me that the food I eat on a typical day is high in fiber and roughage.	0.901			
Attitude				
I think that 3D food printing is generally good.	0.901	0.917	0.941	0.800
I think that 3D food printing is generally important.	0.865			
I think that 3D food printing is generally to be supported.	0.907			
I think that 3D food printing is generally positive.	0.904			
Social norms				
Most people who are important to me think that I should be open to 3D printed food	0.834	0.900	0.927	0.684
I might feel social pressure to buy 3D printed food	0.777			
The people who I listen to could influence me purchasing 3D-printed food	0.889			
My family would support my decision to purchase of 3D printed food	0.884			
My friends would support my decision to purchase 3D-printed food	0.851			
My partner would support my decision to purchase 3D-printed food	0.847			
Capacity				
I believe I have the ability to purchase 3D-printed food	0.900	0.957	0.966	0.824
I see myself as being capable of purchasing 3D-printed food	0.895			
I am confident are I will be able to purchase 3D-printed food	0.929			
For me to purchase 3D-printed food would be easy	0.917			
If I wanted to, I could easily purchase 3D-printed food	0.882			
I am certain that I can purchase 3D-printed food	0.923			
Autonomy				
Whether or not I purchase 3D-printed food is completely up to me	0.863	0.924	0.946	0.814
I would have complete control over whether I purchase 3D-printed food	0.851			
I feel in complete control over whether I will purchase 3D-printed food	0.855			
It is mostly up to me whether or not I purchase 3D-printed food	0.864			
Intention				
I intend to buy 3D printed food when it becomes easily available	0.912	0.950	0.962	0.836
I will buy 3D printed food in the near future	0.944			
Whenever I need to buy food, it is very likely that I would purchase 3D printed food if it was easily available	0.838			
There is a strong possibility that I would purchase 3D printed food	0.947			
I am likely to purchase 3D printed food	0.926			

α, Cronbach’s alpha value; AVE, average variance extracted; CR, composite reliability.

Prior food consumption research demonstrates that behavioral drivers of intention vary substantially depending on product type, cultural context, and the degree of novelty involved. For example, Muflih and Juliana (29) found that spirituality did not directly influence halal-labeled food shopping behavior; instead, its effect was mediated through image, trust, and satisfaction. This suggests that even strongly value-laden food choices are often shaped indirectly through cognitive and affective evaluations rather than through abstract values alone. Similarly, Juliana et al. (30), in their study of Generation Z Muslims using the ShopeeFood platform, they reported that subjective norms and perceived behavioral control significantly predicted purchase intention, while attitude—although rated highly—did not emerge as a significant driver. These findings contrast with studies on novel food technologies, including 3D-printed food, in which attitudes and perceived benefits frequently dominate the formation of intention, while social norms exhibit mixed or context-specific effects. Such inconsistencies highlight that behavioral mechanisms are not universal but rather depend on whether food choices are guided by established norms (e.g., halal compliance) or by perceptions related to uncertainty and innovation. Consequently, the present study adopts the Theory of Planned Behavior and the Value–Attitude–Behavior hierarchy, as these frameworks are particularly suited to capturing both rational evaluations (e.g., perceived benefits, health considerations) and value-driven or affective responses (e.g., neophobia, social influence). This integrative theoretical anchoring is especially appropriate for 3D-printed food, which represents a technologically novel and cognitively demanding consumption context, and allows for a more nuanced explanation of generational differences in adoption intention.

## 3 Results and discussion

### 3.1 Demographic profile

As mentioned, 355 valid responses were collected. These responses comprised 187 respondents (52.7%) classified as Generation X and 168 respondents (47.3%) classified as Generation Y. The sample sizes for Generation X (*n* = 187) and Generation Y (*n* = 168) exceed the minimum requirements for multigroup analysis in PLS-SEM, which recommends at least 100 observations per group or compliance with the 10-times rule based on the maximum number of structural paths pointing to an endogenous construct (31–33). The majority of respondents identified as female (193, 54%), with the remaining as male (162, 46%). Most respondents held a Bachelor’s degree (176, 50%), and 194 (55%) were employed full-time.

### 3.2 Reliability and validity

The reliability and validity of the measurement model were assessed following established guidelines for structural equation modeling in SmartPLS (31, 34). As shown in Table 1, internal consistency reliability was evaluated using both Cronbach’s alpha (α) and composite reliability (CR). The Cronbach’s alpha values for all constructs exceeded the minimum threshold of 0.70 suggested by Narayanamurthy and Tortorella (35), indicating strong internal consistency among the items measuring each latent construct. The Cronbach alpha values ranged from 0.795 to 0.948, suggesting that the construct items reliably captured the intended theoretical domains. Complementing the alpha scores, composite reliability (CR) values ranged from 0.807 to 0.966 across constructs. These values exceed the commonly accepted benchmark of 0.70, confirming high internal consistency and reliability within the constructs (31, 36).

To establish convergent validity, the average variance extracted (AVE) for each construct was examined. As recommended by Fornell and Larcker (34), AVE values above 0.50 indicate that the construct explains more than half the variance in its indicators. All AVE values met or exceeded this threshold, ranging from 0.514 to 0.836, demonstrating satisfactory convergent validity. Furthermore, the high factor loadings for individual items, most of which were well above 0.70, provide further evidence of convergent validity (Table 1), in line with prior 3D food printing literature (15, 16).

Discriminant validity was tested using the heterotrait–monotrait ratio of correlations (HTMT), a robust method for assessing whether constructs are empirically distinct (31, 37). According to the HTMT criterion, values below 0.85 suggest sufficient discriminant validity between constructs. As shown in Table 2, all HTMT values ranged from 0.045 to 0.771, confirming that each construct measures a conceptually distinct domain. Taken together, these results confirm that the measurement model demonstrates strong reliability, adequate convergent validity, and robust discriminant validity. These findings offer confidence in the psychometric quality of the instrument and ensure the structural model analysis can proceed on a sound measurement foundation.

**TABLE 2:** Discriminant validity using the HTMT method.

	ATT	AUT	AWA	BEN	CAP	CON	FOF	FON	HEA	INT	SN
ATT	0.237	0.523	0.410	0.344	0.524	0.045	0.550	0.097	0.135	0.611	
AUT											
AWA	0.569										
BEN	0.713	0.195									
CAP	0.476	0.674	0.504								
CON	0.228	0.495	0.439	0.176							
FOF	0.192	0.085	0.185	0.164	0.177						
FON	0.443	0.127	0.260	0.478	0.128	0.196					
HEA	0.250	0.644	0.422	0.119	0.536	0.513	0.076				
INT	0.771	0.257	0.489	0.672	0.447	0.112	0.274	0.636			
SN	0.698	0.373	0.503	0.535	0.557	0.404	0.214	0.296	0.343		

ATT, attitude; AUT, autonomy; AWA, awareness; BEN, benefits; CAP, capacity; CON, convenience; FOF, food familiarity; FON, food neophobia; HEA, health; INT, intention; SN, social norms.

### 3.3 Hypothesis analysis

Tables 3, 4 present the results of hypothesis testing for each generational cohort using bootstrapped path coefficients, *t*-values, and *p*-values obtained via SmartPLS. Across both generations, awareness, perceived benefits, and attitude consistently emerged as significant predictors of subsequent constructs, demonstrating broad relevance in shaping consumer acceptance of 3D-printed food. For Generation X, awareness had a significant positive influence on attitude (β = 0.165, *p* = 0.000, *f*^2^ = 0.045, indicating a small effect), a finding also evident in the Generation Y group (β = 0.212, *p* < 0.001, *f*^2^ = 0.052, also indicating a small effect). These results suggest that awareness of 3D food printing technology enhances positive attitudes among both generational groups. The effect size is marginally stronger for Generation Y, suggesting that younger consumers may be more responsive to awareness-driven engagement strategies (Tables 3, 4).

**TABLE 3:** Results of the hypotheses testing (Gen X).

Path	Beta	*t*-value	*p*-value	Decision
Awareness → attitude	0.165	5.048	0.000	Supported
Food familiarity → attitude	−0.071	0.071	0.943	Rejected
Food neophobia → attitude	−0.206	3.892	0.000	Supported
Convenience → attitude	0.095	1.315	0.189	Rejected
Health → attitude	0.063	2.068	0.039	Supported
Benefits → attitude	0.518	9.884	0.000	Supported
Attitude → intention	0.623	12.170	0.000	Supported
Social norms → intention	0.042	1.849	0.064	Rejected
Capacity → intention	0.214	1.305	0.189	Rejected
Autonomy → intention	0.045	1.257	0.209	Rejected

**TABLE 4:** Results of the hypotheses testing (Gen Y).

Path	Beta	*t*-value	*p*-value	Decision
Awareness → attitude	0.212	4.154	0.000	Supported
Food familiarity → attitude	0.005	0.105	0.917	Rejected
Food neophobia → attitude	−0.164	2.856	0.004	Supported
Convenience → attitude	0.064	1.001	0.317	Rejected
Health → attitude	0.104	1.797	0.072	Rejected
Benefits → attitude	0.499	7.768	0.000	Supported
Attitude → intention	0.580	9.096	0.000	Supported
Social norms → intention	0.158	2.004	0.045	Supported
Capacity → intention	0.019	0.253	0.800	Rejected
Autonomy → intention	0.112	1.595	0.111	Rejected

The perceived benefits of 3D-printed food consistently demonstrated a strong influence on attitude across both cohorts, with Generation X showing a path coefficient of β = 0.518 (*p* < 0.001, *f*^2^ = 0.198, medium effect) and Generation Y showing a path coefficient of β = 0.499 (*p* < 0.001, *f*^2^ = 0.185, medium effect). This finding confirms that, regardless of age, consumers are more likely to form favorable attitudes when they perceive 3D-printed food as beneficial in terms of convenience, personalization, or sustainability. The similarity of effect sizes in both cohorts underscores the universal appeal of utilitarian and health-related messaging in promoting food innovation.

Notably, the relationship between food neophobia and attitude displayed generational similarities. Among Generation Y, the effect was negative and statistically significant (β = −0.164, *p* = 0.004, *f*^2^ = 0.061, indicating a small effect), suggesting that food neophobia hinders the development of a positive attitude toward 3D-printed food. Similarly, Generation X also revealed a significant negative relationship with a stronger effect size (β = −0.206, *p* = 0.000, *f*^2^ = 0.076, small-to-medium effect). This suggests that food neophobia is a significant barrier to positive attitudes toward 3D-printed foods, regardless of age, although the effect size was slightly larger among Generation X consumers. This implies that targeted information campaigns and positive exposure to 3D-printed food would be important for both age groups when introducing 3D-printed food (Table 4).

Social norms also produced a generationally specific effect. Among Generation Y, social influence significantly impacted their intention to consume 3D-printed food (β = 0.158, *p* = 0.045, *f*^2^ = 0.049, small effect), whereas the same path was not statistically significant for Generation X (β = 0.042, *p* = 0.064, *f*^2^ = 0.008, trivial effect). This result highlights the greater influence of peer and reference group opinions on the food-related choices of younger consumers, consistent with prior literature that characterizes Millennials and younger cohorts as more socially connected and attuned to collective trends. Marketing campaigns that leverage influencers, peer testimonials, or community endorsements may therefore be particularly effective for this group.

Health consciousness significantly influenced attitude toward 3D-printed food among Generation X (β = 0.063, *p* = 0.039, *f*^2^ = 0.026, small effect), but not among Generation Y (β = 0.104, *p* = 0.072, *f*^2^ = 0.022, small effect). While the effect size for Generation X is relatively small, the result suggests that older consumers may be more attentive to the nutritional and wellbeing implications of adopting food innovations, such as 3D printing. In contrast, the absence of a significant relationship for Generation Y may reflect a tendency to prioritize other considerations, such as benefits and social norms, over traditional health motivations when evaluating emerging food technologies. This implies that within the Value–Attitude–Behavior hierarchy, values such as health do not always translate into behavioral intention when alternative values dominate decision-making. This generational divergence aligns with the Theory of Planned Behavior, which posits that the relative importance of attitudinal beliefs varies across different population segments (18). Overall, these findings suggest that health consciousness is a generation-contingent predictor rather than a universal driver of acceptance of 3D-printed food. However, it is worth noting that these differences may not be unique to 3D-printed food. Similarly, Makowska et al. (11), found generational distinctions in general food-related health considerations, suggesting that broader age-related perspectives on nutrition and health may influence consumers’ evaluations. This implies that the differences observed between Generation X and Generation Y in this study are likely driven by their underlying generational health orientations rather than by the specific context of 3D-printed food alone.

The generational differences provide insight into developing communication strategies that resonate with both Generation X and Y consumers. As Generation X consumers’ attitudes were shaped by their health consciousness, communication aimed at this cohort should emphasize nutritional value, quality, and safety. In contrast, for Generation Y consumers, perceived benefits and social norms were more prevalent factors, suggesting that communication focused on innovation, convenience, and peer support would be more appropriate for this group. These results suggest that messaging focused on the health benefits of 3D-printed food (e.g., customizable nutrition, dietary alignment) may be more persuasive for older consumers, whereas younger consumers may require engagement strategies rooted in social influence, sustainability, or experiential value.

Food familiarity, convenience orientation, capacity, and autonomy were not significant predictors of attitude or intention in either group. These findings suggest that when it comes to 3D-printed food — a novel and emerging technology — traditional food consumption motivators may play a secondary role to innovation-specific perceptions such as awareness, benefit perception, and social influence. The non-significant effects of food familiarity, convenience orientation, capacity, and autonomy suggest that traditional food-choice drivers may be attenuated in the context of highly novel food technologies, such as 3D-printed food. From a theoretical perspective, the Theory of Planned Behavior and Diffusion of Innovation indicate that when consumers evaluate unfamiliar technologies, intention formation is driven less by routine-based considerations and more by attitudinal beliefs, perceived benefits, and uncertainty-reducing mechanisms. In this study, the novelty and limited market presence of 3D-printed food likely reduced the relevance of habitual consumption factors, explaining their weak predictive power. Similar attenuation of convenience and familiarity effects has been reported in studies on cultured meat (38) and insect-based foods (39), where innovation-specific perceptions outweigh traditional food decision heuristics. These findings, therefore, challenge assumptions of universal applicability of conventional food choice models and underscore the need for innovation-sensitive extensions when examining emerging food technologies.

Finally, attitude remained a powerful predictor of intention in both groups, reinforcing its central role in the adoption process. For Generation X, the path coefficient was β = 0.623 (*p* < 0.001, *f*^2^ = 0.412, large effect), while Generation Y showed a similarly strong effect (β = 0.580, *p* < 0.001, *f*^2^ = 0.365, large effect). This underscores the robustness of the attitude-intention relationship across age demographics and aligns with the theoretical underpinnings of the Theory of Planned Behavior and related consumer acceptance models. To assess the predictive relevance of the model, Q^2^ values were assessed. For the Generation X cohort, the Q^2^ values for attitude and intention were 0.286 and 0.245, respectively, indicating a medium level of predictive relevance for both. The Generation Y cohort Q^2^ values for attitude and intention were 0.218 and 0.215, respectively, also reflecting acceptable predictive relevance. Therefore, the findings reveal that the model predicts both attitude and intention across both generational cohorts.

The differences observed between Generation X and Generation Y in this study can be attributed to the distinct socio-cultural, technological, and experiential factors that shape each cohort’s perceptions of food innovation. Generation X consumers, who generally have more established food preferences and greater caution toward unfamiliar technologies, tend to rely more on factors such as health consciousness and reduced neophobia when forming attitudes toward 3D-printed food. In contrast, Generation Y—who grew up during rapid technological advancement and are more accustomed to digital innovation—are more strongly influenced by perceived benefits, social norms, and awareness-driven cues. Their greater exposure to social media, online communities, and peer-driven trends likely heightens the role of social influence in their decision-making. These generational distinctions suggest that attitudes toward 3D-printed food are shaped not only by individual psychological factors but also by broader generational experiences with technology, health, and social communication, resulting in differing pathways to adoption across age groups.

In addition to generational differences in attitudes and perceptions, recent research in Human-Food Interaction (HFI) has highlighted the potential of interactive, digitally augmented food experiences to influence acceptance of 3D-printed foods. For instance, Miyatake and Punpongsanon (40) introduced EateryTag, an unobtrusive edible tagging system that embeds digital tags within food, preserving its esthetics and sensory qualities while enabling the retrieval of digital information. This study demonstrates how 3D printing can seamlessly integrate technology into the dining experience, enhancing engagement without compromising culinary integrity. Similarly, Lin et al. (41) developed FoodFab, a 3D food printing system that creates perceptual illusions to modify chewing time and perceived satiety by adjusting infill patterns and density. Their work illustrates how structural and sensory manipulations via 3D printing can shape personalized eating experiences, linking technological innovation to user satisfaction and consumption behavior. In another example, Zoran and Cohen (42) proposed a modular mold system for programmable taste structures, allowing chefs to control flavor distribution and efficiently create a variety of shape permutations. This approach combines digital tools with culinary craftsmanship, demonstrating that digital gastronomy can enrich user interaction and experiential value. Collectively, these HFI studies indicate that beyond cognitive awareness, perceived benefits, and health considerations, experiential factors such as interactive, sensory, and personalization features can significantly influence consumer attitudes and intentions toward 3D-printed foods. Such insights are particularly relevant for younger, tech-savvy consumers, like Generation Y, who may respond more positively to innovations that integrate digital interactivity with taste and presentation.

The current study advances existing research on 3D-printed food acceptance by both reinforcing and extending prior findings. Consistent with earlier studies [e.g., Ng et al. (15) Brunner et al. (16)], awareness, perceived benefits, and attitude remained central drivers of consumer acceptance; however, this study contributes new insight by demonstrating how these relationships differ between Generation X and Generation Y within the underexplored South African context. The findings also deepen understanding of food neophobia by showing that its negative influence persists across generations, with a slightly stronger effect among older consumers, an aspect not previously highlighted in generational comparisons. Moreover, the study challenges assumptions in earlier literature (8) that social norms uniformly influence technology-related food choices, revealing that such effects are significant for Generation Y but not for Generation X. Finally, the observed generational differences in the role of health consciousness suggest that the motivations underlying the acceptance of 3D-printed food may be more nuanced than previously documented. Collectively, these contributions refine theoretical expectations and provide a more context-specific understanding of how consumers engage with emerging food technologies.

Cross-country comparisons further highlight the distinct contribution of this study. While prior studies conducted in high-income and technologically mature markets—such as Europe, North America, and East Asia—generally report stronger and more uniform effects of perceived benefits, social norms, and technological enthusiasm on acceptance of 3D-printed food (7, 8), the present findings reveal a more differentiated pattern within the South African context. In particular, the weaker and generation-contingent influence of social norms, alongside the sustained role of food neophobia and health consciousness among older consumers, contrasts with studies from Western contexts where social influence tends to exert broader effects across age groups. This divergence suggests that emerging-market consumers may evaluate food innovations through a more cautious, value-driven lens shaped by socio-economic constraints, cultural food traditions, and uneven exposure to food technologies. By empirically demonstrating that the relative salience of behavioral drivers varies not only by generation but also by national context, this study extends the 3D food printing literature beyond predominantly Global North perspectives and underscores the importance of context-sensitive models of food technology acceptance.

Importantly, the inconsistencies observed across countries suggest that consumer acceptance of 3D-printed food is not governed by a universal psychological model but is highly contingent on contextual factors, including market maturity, cultural food norms, and technological exposure. In technologically mature contexts, perceived benefits and experiential attributes tend to dominate intention formation, whereas in emerging markets, uncertainty-related factors such as neophobia and trust play a stronger role. The present findings from South Africa align more closely with studies conducted in other emerging or transitional economies (43), reinforcing the argument that acceptance mechanisms are context-sensitive rather than globally uniform. This highlights the limitation of directly generalizing findings from high-income countries and underscores the importance of contextually grounded behavioral models for novel food technologies. Taken together, both significant and non-significant findings indicate that consumer intention toward 3D-printed food is shaped by innovation-specific cognitive and attitudinal mechanisms rather than by habitual food choice factors, particularly in emerging market contexts.

Beyond generational differences, the findings also reflect deeper psychological and cultural dynamics that shape the acceptance of emerging food technologies within the South African context. As a rapidly modernizing society with increasing exposure to digital and technological innovation, South Africa presents a unique environment where curiosity about novel technologies coexists with cautious attitudes toward unfamiliar foods (3). Psychological factors, such as risk perception, trust in technology, and openness to innovation, likely interact with historical food norms and culturally rooted expectations surrounding naturalness and traditional preparation methods. These cultural orientations may influence how consumers evaluate the safety, authenticity, and desirability of 3D-printed foods, particularly in contexts where food plays a significant social and communal role. Additionally, South Africa’s technological landscape characterized by uneven access, varying levels of digital literacy, and differing degrees of exposure to global food trends, may contribute to divergent responses across consumer groups. Taken together, these technological, psychological, and cultural dynamics suggest that acceptance of 3D-printed food in South Africa is shaped not only by individual-level attitudes but also by broader socio-cultural and technological forces that merit deeper exploration in future research.

In summary, the results from Tables 3, 4 demonstrate both shared and distinct pathways influencing the intentions of Generations X and Y to consume 3D-printed food. These insights hold valuable implications for segmentation and targeted messaging in the commercialization of food printing technology. While awareness and benefit perceptions remain universally important, social and psychological nuances such as neophobia and social norms should be considered when crafting age-sensitive adoption strategies.

### 3.4 Practical, policy, and sustainability implications

The present findings offer several practical and policy-relevant implications for the responsible diffusion of 3D food printing within emerging food systems such as South Africa. From a policy perspective, the observed generational differences in adoption drivers highlight the need for regulatory frameworks that go beyond food safety compliance to also address consumer trust, transparency, and nutritional functionality. As 3D-printed foods fall within the category of novel or digitally enabled foods, regulators may need to clarify approval pathways, ingredient disclosure requirements, and labeling standards that communicate both nutritional value and production processes. Integrating such considerations into national food innovation and food safety policies could facilitate public confidence and accelerate responsible market entry.

To operationalize these implications, food authorities could introduce dedicated regulatory pathways for 3D-printed and digitally enabled foods, similar to novel food or regulatory sandbox frameworks, allowing controlled market entry and evaluation. Clear labeling standards that specify production methods, nutritional customization, and sustainability attributes would enhance transparency and trust, particularly for health-conscious consumers. In parallel, public–private innovation programs could support pilot deployments in institutional settings (e.g., hospitals and universities) that align 3D food printing with national nutrition goals, food waste reduction, and responsible consumption objectives.

From an industry standpoint, the findings suggest that commercialization strategies for 3D-printed foods should be segmented by generational profiles rather than relying on uniform market approaches. For older consumers, whose intentions are more strongly shaped by health consciousness, firms may benefit from emphasizing nutritional personalization, functional ingredients, and clinically relevant benefits through clear labeling and evidence-based health communication. In contrast, younger consumers’ sensitivity to social norms implies that adoption may be enhanced through experiential marketing, social proof, and visibility within social dining, hospitality, and digital food-service environments. Early diffusion through controlled contexts such as restaurants, institutional catering, hospitals, or university campuses may also help normalize the technology and reduce food neophobia across age groups.

## 4 Limitations and future research agenda

While this study provides novel insights into generational differences in intentions to consume 3D-printed food in South Africa, several limitations also highlight important directions for future research. First, although the sample size was adequate for PLS-SEM analysis, respondents were limited to individuals with prior awareness of 3D-printed food. This purposive approach ensured informed responses but introduced self-selection bias and constrained generalizability. Future studies should adopt staged designs that include both unaware and aware consumers to examine how exposure, education, and information framing dynamically shape acceptance over time. Incorporating expert stakeholders—such as food technologists, nutritionists, and policymakers—would further enrich understanding of supply- and regulation-side perspectives.

Second, the reliance on an online, self-administered questionnaire may have favored digitally literate and technology-inclined participants. Future research could strengthen external validity by using mixed-methods approaches, including in-person surveys, qualitative interviews, and community-based sampling, particularly with underrepresented or rural populations. Third, the cross-sectional design limits causal inference and does not capture how consumer perceptions evolve as 3D food printing becomes more visible in real-world contexts. Longitudinal or repeated cross-sectional studies are therefore recommended to track shifts in awareness, neophobia, trust, and consumption intention as market exposure increases.

Importantly, future research should move beyond attitudinal intentions toward experiential and behavioral validation. Integrating sensory science methods—such as tasting trials, texture and appearance evaluations, and consumer hedonic testing—would provide more ecologically valid insights into acceptance mechanisms. Field experiments in food service settings (e.g., hospitals, schools, or personalized nutrition programs) could further examine real consumption behavior, willingness to pay, and post-consumption satisfaction.

Finally, given the increasing role of digitalization in food systems, future studies could explore AI-driven and data-enabled food personalization within 3D food printing, particularly how algorithm-based nutrition customization influences trust, perceived benefits, and ethical acceptance across generations. Examining these dimensions would not only extend behavioral theory but also align future research with broader agendas in nutrition sustainability, personalized nutrition, and smart food systems. Additionally, the present study did not include explicit measures of sustainability perception or ethical orientation, both of which are theoretically relevant to consumer acceptance of novel food technologies and may differ meaningfully across generational cohorts. Future research should incorporate these dimensions to provide a more complete account of the values-based drivers of intention, particularly given the sustainability narratives that increasingly frame 3D-printed food in public and policy discourse.

## 5 Conclusion

This study provides insights into generational differences in the adoption of 3D-printed food in South Africa, showing that awareness, perceived benefits, and attitudes are key drivers of consumption intention for both Generation X and Generation Y. Generational differences were observed in the influence of food neophobia, social norms, and health consciousness, suggesting that some determinants are universal, while others are cohort-specific. These findings underscore the importance of age-sensitive communication strategies, including health-focused messaging for older consumers and socially driven approaches for younger consumers, as well as the role of education and experiential exposure in reducing neophobia.

Beyond documenting generational differences, this study is among the first to empirically demonstrate that the psychological mechanisms underlying 3D-printed food adoption differ across age cohorts in an emerging-market context, thereby challenging the assumption of uniform consumer responses embedded in existing food-technology acceptance models.

Theoretically, this study extends existing models of technology acceptance and food innovation by demonstrating that generational context moderates the pathways through which consumers form intentions toward emerging food technologies. Specifically, it reveals that social influence is more salient for younger consumers, while health consciousness strongly influences the intentions of older consumers, underscoring the need to integrate demographic moderators into frameworks such as the Theory of Planned Behavior and the Diffusion of Innovation. By revealing these generational distinctions, the study provides a novel understanding of how consumer adoption of emerging foods varies across age cohorts and lays the foundation for future research on tailored behavioral models for these technologies.

## Data Availability

The original contributions presented in this study are included in this article/supplementary material, further inquiries can be directed to the corresponding author.
